# The Complement System Contributes to Functional Antibody-Mediated Responses Induced by Immunization with Plasmodium falciparum Malaria Sporozoites

**DOI:** 10.1128/IAI.00920-17

**Published:** 2018-06-21

**Authors:** Marije C. Behet, Liriye Kurtovic, Geert-Jan van Gemert, Celine M. Haukes, Rianne Siebelink-Stoter, Wouter Graumans, Marga G. van de Vegte-Bolmer, Anja Scholzen, Jeroen D. Langereis, Dimitri A. Diavatopoulos, James G. Beeson, Robert W. Sauerwein

**Affiliations:** a Radboud University Medical Center, Department of Medical Microbiology, Nijmegen, The Netherlands; b Burnet Institute, Melbourne, Australia; c Department of Immunology and Pathology, Monash University, Melbourne, Australia; d Laboratory of Pediatric Infectious Diseases, Radboud University Medical Center and Radboud Center for Infectious Diseases, Nijmegen, The Netherlands; e Department of Medical Microbiology, Monash University, Clayton, Australia; University of South Florida

**Keywords:** chemoprophylaxis, sporozoites, IgG, Plasmodium falciparum, antibodies, complement, controlled human malaria infection, immunization, liver stage, sporozoites

## Abstract

Long-lasting and sterile homologous protection against malaria can be achieved by the exposure of malaria-naive volunteers under chemoprophylaxis to Plasmodium falciparum-infected mosquitoes (chemoprophylaxis and sporozoite [CPS] immunization). While CPS-induced antibodies neutralize sporozoite infectivity *in vitro* and *in vivo*, antibody-mediated effector mechanisms are still poorly understood. Here, we investigated whether complement contributes to CPS-induced preerythrocytic immunity. Sera collected before and after CPS immunization in the presence of active or inactive complement were assessed for the recognition of homologous NF54 and heterologous NF135.C10 sporozoites, complement fixation, sporozoite lysis, and possible subsequent effects on *in vitro* sporozoite infectivity in human hepatocytes. CPS immunization induced sporozoite-specific IgM (*P* < 0.0001) and IgG (*P* = 0.001) antibodies with complement-fixing capacities (*P* < 0.0001). Sporozoite lysis (*P* = 0.017), traversal (*P* < 0.0001), and hepatocyte invasion inhibition (*P* < 0.0001) by CPS-induced antibodies were strongly enhanced in the presence of active complement. Complement-mediated invasion inhibition in the presence of CPS-induced antibodies negatively correlated with cumulative parasitemia during CPS immunizations (*P* = 0.013). While IgG antibodies similarly recognized homologous and heterologous sporozoites, IgM binding to heterologous sporozoites was reduced (*P* = 0.023). Although CPS-induced antibodies did not differ in their abilities to fix complement, lyse sporozoites, or inhibit the traversal of homologous and heterologous sporozoites, heterologous sporozoite invasion was more strongly inhibited in the presence of active complement (*P* = 0.008). These findings demonstrate that CPS-induced antibodies have complement-fixing activity, thereby significantly further enhancing the functional inhibition of homologous and heterologous sporozoite infectivity *in vitro*. The combined data highlight the importance of complement as an additional immune effector mechanism in preerythrocytic immunity after whole-parasite immunization against Plasmodium falciparum malaria.

## INTRODUCTION

Malaria is one of the world's chief causes of morbidity and mortality by infectious diseases and has a significant impact on public and economic health worldwide. Nearly half of the world's population is at risk of malaria, and in 2015, there were roughly 200 million clinical cases and nearly half a million deaths attributed to malaria (1). Malaria is caused by the protozoan parasite Plasmodium and is characterized by a complex multistage life cycle in the human host. Sporozoites deposited into the skin by a female Plasmodium falciparum-infected Anopheles mosquito first travel to the liver by gliding motility (2) and cross cell barriers by breaching host cell membranes (3). After an invasion-and-maturation step in the liver, merozoites progress to invade erythrocytes, leading to the clinical symptoms of malaria (4). Although a significant decrease in malaria mortality rates has been observed in the last 15 years (1), malaria control efforts are threatened by the emergence of drug-resistant parasites (5) and insecticide-resistant mosquitoes (6), stressing the need for a highly effective vaccine.

While achieving sterile immunity by subunit vaccination has proven to be difficult (7–9), long-lasting and sterile protection against a homologous P. falciparum malaria infection can be accomplished experimentally by whole-parasite immunization with live attenuated sporozoites. For instance, this can be achieved in healthy human volunteers by the intravenous injection of 150,000 cryopreserved sporozoites (PfSPZ-CVac) (10) or by exposure to bites of 30 to 45 P. falciparum-infected mosquitoes under chloroquine chemoprophylaxis (chemoprophylaxis and sporozoites [CPS]) (11, 12). This immunity is long-lasting, involving effector memory T-cell responses as well as memory B-cell and antibody responses recognizing preerythrocytic-stage antigens (12–17). CPS-induced antibodies show neutralizing activity against sporozoite and liver-stage parasites and are capable of reducing liver-stage infection in hepatocytes *in vitro* and *in vivo* in a human liver-chimeric mouse model (18). CPS-induced antibodies show a much stronger effect on reducing liver-stage infection *in vivo* than *in vitro*, suggesting that additional effector mechanisms besides the direct neutralization of sporozoites by antibodies may be involved.

One possible mechanism is the activation of complement, representing a system of heat-sensitive, soluble, and cell surface-associated proteins that are involved in pathogen opsonization, the recruitment of phagocytes, and pathogen lysis via downstream C3 complement protein deposition (19). The complement pathway plays a key role in the antibody-mediated inhibition of P. falciparum merozoite invasion, thereby reducing P. falciparum blood-stage replication and preventing clinical disease (20). Additionally, P. falciparum sporozoites are susceptible to complement activation by human antibodies that are naturally acquired after exposure to multiple infections in areas where malaria is endemic. Naturally acquired antibodies are able to promote complement deposition and activation, resulting in enhanced antibody-mediated traversal inhibition *in vitro* (21). Here, we studied whether antibody-dependent complement activation contributes to preerythrocytic-antibody-mediated protective immunity against P. falciparum malaria sporozoites induced by CPS immunization. To this end, CPS-induced antibodies were assessed for their functional capacity to fix complement proteins on sporozoites, induce sporozoite lysis, and further impact *in vitro* hepatocyte traversal and invasion by sporozoites in the presence of active complement, tested for both the homologous NF54 strain the genetically and geographically distinct NF135.C10 parasite clone.

## RESULTS

### CPS immunization induces sporozoite-specific IgG and IgM antibodies.

**FIG 1:**
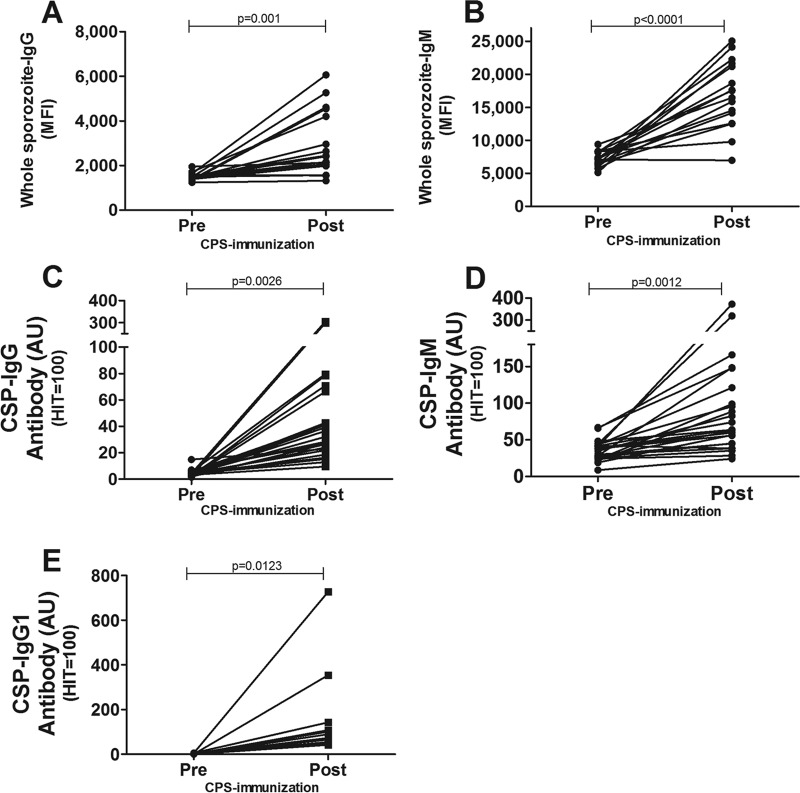
Recognition of homologous P. falciparum NF54 sporozoites by CPS-induced antibodies. Homologous NF54 sporozoites were preincubated with 10% inactive complement and 10% heat-inactivated pre- or postimmunization serum from CPS-immunized volunteers (*n* = 16). (A and B) The amounts of CPS-induced IgG (A) and IgM (B) antibodies recognizing sporozoites were determined by flow cytometry and are shown as geometric mean fluorescence intensities (MFI). (C and D) Levels of IgG (C) and IgM (D) antibodies to CSP before (Pre) and after (Post) completed CPS immunization in CPS-immunized volunteers (*n* = 24) were determined by ELISAs and are shown as arbitrary units (AU), as defined by serial dilutions of a reference standard serum pool with high antibody concentrations. (E) CSP-specific IgG1 antibodies in pre- and postimmunization samples from CPS-immunized volunteers (*n* = 15) were determined by CSP-specific IgG1 subclass ELISAs and are shown as arbitrary units. Differences between pre- and postimmunization samples were determined by paired Student's *t* test, and a *P* value of <0.05 was considered statistically significant.

Sporozoite-specific IgG antibodies were specifically induced in 15 out of 16 volunteers after completed CPS immunization using NF54-infected mosquitoes, with a median fold increase of 1.6 and an interquartile range (IQR) of 1.2 to 3.1 (*P* < 0.0001 [Fig. 1A] and *P* = 0.004 [see Fig. S1A and S1C in the supplemental material]) compared to the baseline. CPS immunization also strongly induced sporozoite-specific IgM antibodies in 15 out of 16 volunteers, with a median fold increase of 2.3 (IQR, 1.8 to 2.9; *P* < 0.0001 [Fig. 1B] and *P* < 0.0001 [Fig. S1B and S1D]). There was no correlation between IgG and IgM antibodies (*P* = 0.192) (Fig. S2A). Specific IgG and IgM antibodies against the dominant circumsporozoite protein (CSP) were induced (*P* = 0.0026 and *P* = 0.0012) (Fig. S1C and S1D) and were positively correlated (*P* = 0.0194) (Fig. S2B). While whole-sporozoite-specific IgG antibodies did not correlate with anti-CSP-specific IgG (anti-CSP-IgG) antibody levels (*P* = 0.269) (Fig. S2C), sporozoite-specific IgM antibodies correlated with anti-CSP-IgM antibody levels (*P* = 0.003) (Fig. S2D).

### IgG1 and IgM isotype antibodies to CSP are most prevalent in CPS-immunized volunteers.

Antibody isotype is a major factor in the subsequent activation of the classical complement pathway. Levels of both anti-CSP-specific IgM (*P* = 0.0012) (Fig. 1D) and IgG1 (*P* = 0.0123) (Fig. 1E) antibodies were significantly increased, while IgG2, IgG3, and IgG4 antibodies against CSP remained undetectable after CPS immunization (data not shown). Anti-CSP-IgG1 levels strongly correlated with total CSP-specific IgG, confirming that CSP-IgG1 antibodies are primarily induced in CPS-immunized volunteers (*P* = 0.0001) (Fig. 1E). The combined data demonstrate that CPS immunization predominantly induces IgG1 and IgM antibodies, both of which are known to be potent activators of the complement pathway (22).

### CPS-induced antibodies fix complement and lyse homologous P. falciparum NF54 sporozoites.

**FIG 2:**
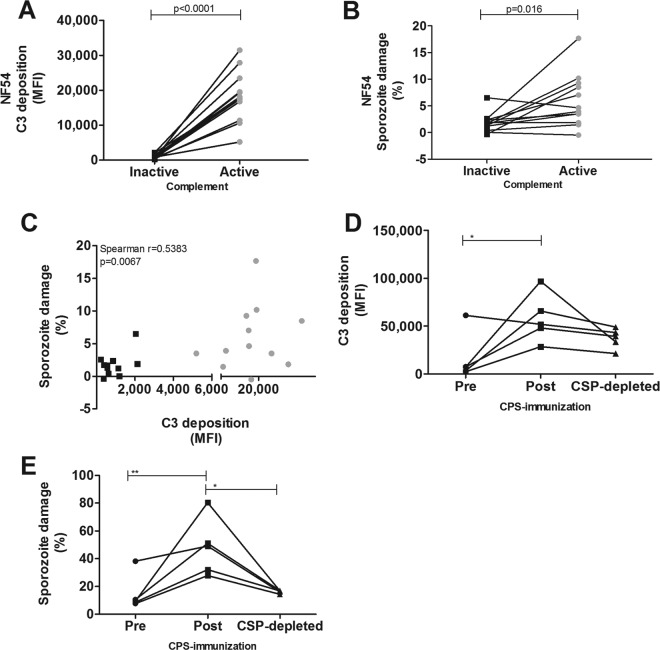
Complement activation and lysis of homologous P. falciparum NF54 sporozoites by CPS-induced antibodies. Homologous NF54 sporozoites were preincubated with 10% inactive or active complement and 10% heat-inactivated pre- or postimmunization serum from CPS-immunized volunteers. (A) C3 complement protein deposition on NF54 sporozoites in 10% postimmunization serum (*n* = 12 volunteers) in the presence of inactive or active of complement was assessed and is shown as MFI. C3 deposition by postimmunization serum was corrected for baseline responses by subtracting C3 deposition by that for preimmunization serum. (B) Sporozoite damage by CPS-induced antibodies (*n* = 12 volunteers) in the presence of 10% inactive or active complement, shown as percent sporozoite damage and corrected by subtracting the percent sporozoite damage in the presence of preimmunization antibodies. (C) Scatter plots showing C3 complement protein deposition. The percent damaged sporozoites per donor was corrected for preimmunization values and analyzed by Spearman correlation analysis (*n* = 12 CPS-immunized volunteers). Samples in the presence or absence of active complement are shown with gray circles and black squares, respectively. (D and E) NF54 sporozoites were preincubated with 10 mg/ml of purified preimmunization IgGs, postimmunization IgGs, and postimmunization IgGs depleted from CSP-specific antibodies (*n* = 5 volunteers) in the presence of 10% active complement. C3 complement protein deposition (D) and sporozoite damage by purified IgGs (E) are shown as C3 deposition (MFI) and the percentage of sporozoite damage, respectively. Comparisons between multiple groups were performed by one-way ANOVA with a Bonferroni multiple-comparison *post hoc* test. Data are shown as the means of results from duplicate measurements and presented as black squares or gray circles for samples tested in the presence of inactive or active complement, respectively. Asterisks represent *P* values of <0.05 (*) and <0.01 (**).

C3 complement protein deposition on sporozoites and sporozoite membrane permeability were strongly enhanced in the presence of postimmunization antibodies and active complement (*P* < 0.0001 and *P* = 0.016) (Fig. 2A and B). As expected, there was a strong correlation between C3 deposition and sporozoite membrane permeability, suggesting that antibody-dependent C3 deposition on sporozoites results in functional sporozoite lysis (*P* = 0.0067) (Fig. 2C).

Activation of the complement pathway can also occur via antibody-independent pathways. Indeed, C3 deposition and sporozoite lysis also occurred in the presence of preimmunization serum, with median fold increases of 12.2 (IQR, 10.2 to 13.7) and 1.30 (IQR, 1.1 to 1.6), respectively (*P* < 0.0001 and *P* = 0.0009) (see Fig. S3A and S3B in the supplemental material). However, this effect was much weaker than that in the presence of CPS-induced antibodies (*P* < 0.0001 and *P* = 0.002) (Fig. S3C and S3D), with median fold increases of 16.2 (IQR, 14.5 to 20.0) and 1.8 (IQR, 1.4 to 2.2). Thus, complement activation against sporozoites via the antibody-dependent classical pathway is more potent than those via antibody-independent pathways.

We next investigated to which degree complement activation is mediated by anti-CSP-specific IgG antibodies. C3 deposition on sporozoites and sporozoite lysis were enhanced in the presence of postimmunization rather than preimmunization IgG (*P* < 0.05 and *P* < 0.01) (Fig. 2D and E). The level of sporozoite lysis in the presence of anti-CSP-depleted postimmunization IgG was also significantly lower than that in the presence of postimmunization IgG (*P* < 0.05) (Fig. 2E). The combined data suggest that anti-CSP-IgG antibodies contribute to the functional CPS-induced complement-mediated antibody response but also show that antibodies to other sporozoite surface-expressed proteins may be involved as well.

### Complement-dependent inhibition of homologous P. falciparum NF54 sporozoite infectivity.

**FIG 3:**
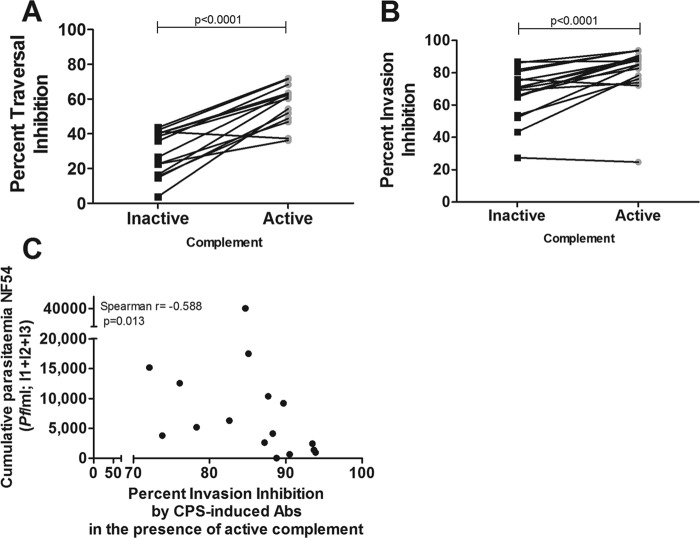
*In vitro* traversal and invasion inhibition of homologous P. falciparum NF54 sporozoites by CPS-induced antibodies in the presence or absence of complement. (A and B) The percent inhibition of traversal (14 volunteers) (A) and invasion (17 volunteers) (B) was calculated for 10% postimmunization serum compared to preimmunization serum for each volunteer in the presence of 10% inactive or active complement. (C) Spearman correlation analysis between cumulative parasitemia (P. falciparum parasites per milliliter) during three CPS immunizations and invasion inhibition by CPS-induced antibodies (Abs) in the presence of active complement (uncorrected for HIS) (*n* = 17 volunteers). Data are shown as the means of results from duplicate measurements and presented as black squares or gray circles for samples tested in the presence of inactive or active complement, respectively.

Antibody-mediated inhibition of sporozoite traversal was enhanced in the presence of active complement, with median traversal inhibition percentages of 31.2% (IQR, 16.2 to 40.4%) and 60.8% (IQR, 48.3 to 64.6%) for inactive and active complement, respectively (*P* < 0.0001) (Fig. 3A). Similarly, sporozoite invasion was reduced more efficiently in the presence of active complement, with median invasion inhibition percentages of 87.2% (IQR, 77.2 to 90.1%) and 70.1% (IQR, 59.4 to 78.4%) for active and inactive complement, respectively (*P* < 0.0001) (Fig. 3B). Cumulative parasitemia during CPS immunizations negatively correlated with NF54 invasion inhibition *in vitro* by CPS-induced antibodies in the presence of active complement (*P* = 0.013) (Fig. 3C) but not inactive complement (*P* = 0.2518) (see Fig. S4A in the supplemental material).

### Complement-dependent inhibition of heterologous P. falciparum NF135.C10 sporozoite infectivity.

**FIG 4:**
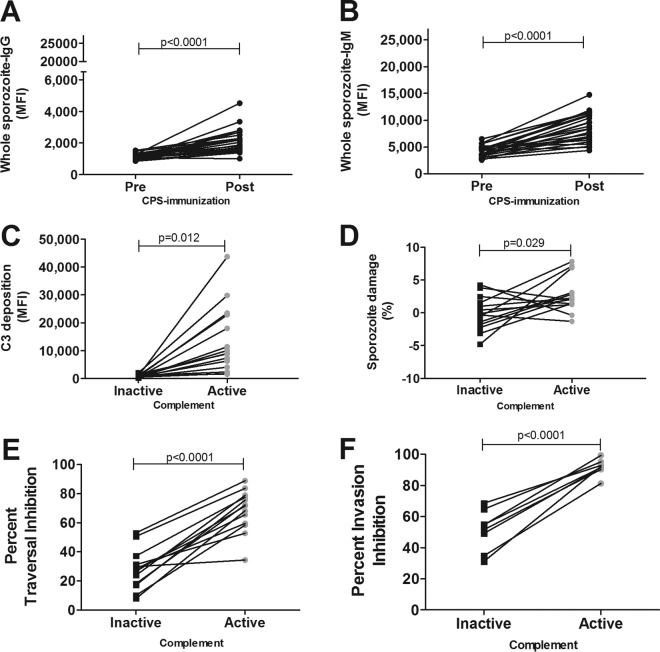
Complement activation and inhibition of heterologous P. falciparum NF135.C10 sporozoites. Heterologous P. falciparum NF135.C10 sporozoites were preincubated with 10% heat-inactivated pre- or postimmunization serum (*n* = 24 volunteers) and 10% inactive complement. (A and B) The amounts of CPS-induced IgG (A) and IgM (B) antibodies recognizing sporozoites were determined by flow cytometry and are shown as MFI. (C) C3 complement deposition on NF135.C10 sporozoites in the presence of 10% inactive (black) or active (gray) complement by 10% pre- or postimmunization CPS serum (*n* = 15 volunteers), shown as the MFI. C3 complement protein deposition in the presence of postimmunization serum was corrected for C3 deposition in the presence of preimmunization serum. (D) Sporozoite damage by pre- or postimmunization antibodies (*n* = 14 volunteers) in the presence of 10% active or inactive complement, shown as the percent sporozoite damage and corrected for the percent sporozoite damage in the presence of preimmunization antibodies. (E and F) The percentages of inhibition of heterologous sporozoite traversal (13 volunteers) (E) and invasion (*n* = 8 volunteers) (F) were calculated for 10% postimmunization serum compared to preimmunization serum for each volunteer in the presence of 10% inactive or active complement. Data are shown as the means of results from duplicate measurements and presented as black squares or gray circles for samples tested in the presence of inactive or active complement, respectively. Differences between pre- and postimmunization samples or inactive and active complement were determined by paired Student's *t* test, and a *P* value of <0.05 was considered statistically significant.

It was shown previously that CPS-induced antibodies can inhibit *in vitro* hepatocyte invasion by sporozoites of the homologous NF54 strain but also the genetically and geographically distinct NF135.C10 clone (23, 24). Therefore, we next examined whether the effects of complement on NF54 sporozoites also extend to heterologous NF135.C10 sporozoites. Both CPS-induced IgG and IgM antibodies recognized NF135.C10 sporozoites (*P* < 0.0001) (Fig. 4A and B). Complement fixation and lysis of heterologous sporozoites were also enhanced by postimmunization antibodies in the presence of active complement (*P* = 0.012 and *P* = 0.029) (Fig. 4C and D). Similar to NF54 sporozoites, heterologous traversal and invasion were inhibited more efficiently by postimmunization antibodies in the presence of active complement (*P* < 0.0001) (Fig. 4E and F). Median traversal inhibition percentages were 71.6% (IQR, 59.0 to 78.2%) and 28.2% (IQR, 17.7 to 34.3%), and median invasion inhibition percentages were 91.8% (IQR, 90.5 to 94.5%) and 53.0% (IQR, 38.4 to 62.2%) for active and inactive complement, respectively. There was no correlation between complement-dependent NF135.C10 invasion inhibition by CPS-induced antibodies *in vitro* and that during the prepatent period following NF135.C10 challenge infection, although only a limited number of volunteers could be tested (*P* = 0.083; *n* = 4 volunteers) (see Fig. S4B in the supplemental material). These combined data suggest that broadly neutralizing and complement-fixing antibodies are also potent against heterologous parasites.

### Comparison of complement-mediated effects on P. falciparum NF54 and NF135.C10 sporozoite infectivity.

**FIG 5:**
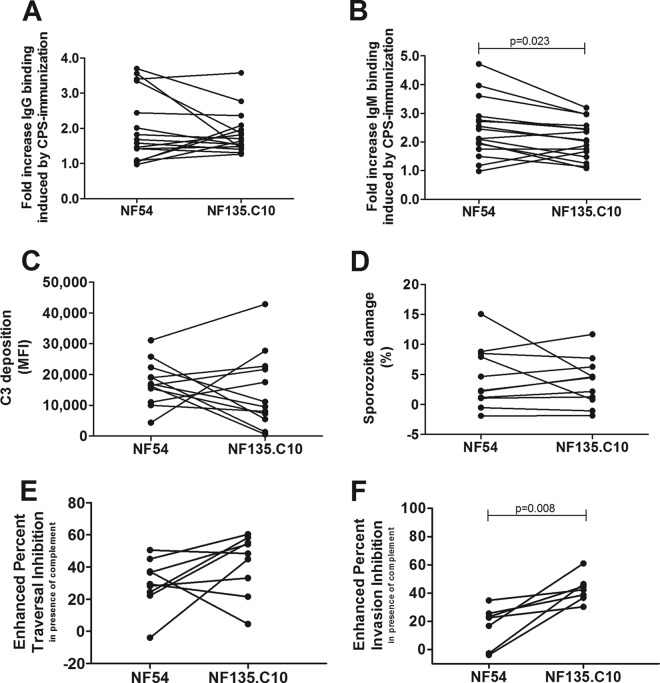
Comparison of complement activation and inhibition between NF54 and NF135.C10 sporozoites. (A and B) Recognition of homologous NF54 and heterologous NF135.C10 sporozoites by postimmunization IgG (A) and IgM (B) antibodies, shown as fold increases over baseline (preimmunization) antibody values (*n* = 16 CPS-immunized volunteers). (C) Enhanced C3 complement protein deposition on homologous and heterologous sporozoites, shown as the MFI and corrected for complement fixation by preimmunization antibodies and inactive complement (*n* = 12 volunteers). (D) Enhanced sporozoite damage of homologous and heterologous sporozoites, shown as percent sporozoite damage and corrected for damage in the presence of preimmunization antibodies and inactive complement (*n* = 11 volunteers). (E and F) The percentages of enhanced inhibition of both homologous and heterologous sporozoite traversal (*n* = 9 volunteers) (E) and invasion (*n* = 7 volunteers) (F) in the presence of active complement were calculated for 10% postimmunization serum compared to preimmunization serum for each volunteer in the presence of 10% inactive or active complement. To calculate the percent enhanced inhibition by active complement and postimmunization antibodies, inhibition in the presence of complement was corrected for inhibition in the presence of inactive complement. Data are shown as the means of results from duplicate measurements. Differences between parasite strains were determined by paired Student's *t* test, and a *P* value of <0.05 was considered statistically significant.

There were no significant differences in the binding of specific IgG antibodies to NF54 and NF135.C10 sporozoites (*P* = 0.494) (Fig. 5A). In contrast, NF135.C10 sporozoites were less opsonized by specific IgM antibodies than were NF54 sporozoites (*P* = 0.023) (Fig. 5B). Nevertheless, this did not translate into differences in C3 deposition and lysis (*P* = 0.53 and *P* = 0.69) (Fig. 5C and D). Traversal inhibition was not significantly different between the two strains (*P* = 0.18) (Fig. 5E), with median percent enhanced traversal inhibition values of 29.4% (IQR, 23.4 to 41.1%) and 48.4% (IQR, 27.3 to 57.7%) for NF54 and NF135.C10 sporozoites, respectively. NF54 sporozoite invasion was neutralized more strongly by CPS-induced antibodies in the absence of active complement, with median percent invasion inhibition values of 70.1% (IQR, 59.4 to 78.4%) and 53.0% (IQR, 38.4 to 62.2%) for NF54 and NF135.C10 sporozoites, respectively (*P* = 0.019). Percent invasion inhibition values for NF54 and NF135.C10 sporozoites in the presence of active complement were not significantly different (medians, 87.2% [IQR, 77.2 to 90.2%] and 91.8% [IQR, 90.5 to 94.5%] for NF54 and NF135.C10, respectively). However, the invasion of heterologous sporozoites in the presence of active complement was inhibited more strongly than was the invasion of NF54 sporozoites (*P* = 0.008) (Fig. 5F), with median enhanced invasion inhibition values of 22.5% (IQR, −2.7 to 25.5%) and 42.3% (IQR, 36.4 to 46.4%) for NF54 and NF135.C10 sporozoites, respectively.

## DISCUSSION

The present study shows that CPS immunization with P. falciparum NF54 sporozoites induces complement-fixing antibodies that are capable of activating the classical complement pathway, resulting in membrane-compromised sporozoites and a further reduction of homologous and heterologous sporozoite infectivity *in vitro*. This is supported by previous findings from *in vitro* assays with Plasmodium gallinaceum revealing the importance of the classical complement pathway in inducing sporozoite death (25).

Sporozoite-specific IgM responses contribute to CPS-induced immunity, with the potential benefits of higher avidity for P. falciparum target antigens and efficient complement fixation (26). Some volunteers tend to have a stronger induction of sporozoite-specific IgM than IgG antibodies, while sporozoite-specific IgG antibodies are induced more strongly in other volunteers. As shown in the present study, CSP- and sporozoite-specific IgM antibodies are still present 18 weeks after the last CPS immunization, corroborating previous observations (27). This suggests that an initial IgM response is developed, whereby not all epitope-specific responses might be followed by class switching to IgG over the next 2 to 3 weeks (28). The functional relevance and importance of IgM antibodies were shown previously in the Plasmodium chabaudi model, where Plasmodium-specific IgM-producing memory B cells were readily induced in response to repeated parasite exposure (29). Interestingly, the magnitude and breadth of P. falciparum-specific IgM antibody responses are also higher in African adults with naturally acquired antimalarial immunity who are resistant to malaria than in adults who are susceptible to malaria (30). Additionally, it was shown very recently that immunization of Tanzanian individuals with radiation-attenuated cryopreserved P. falciparum sporozoites (PfSPZ) induces functional antisporozoite IgM antibodies with human hepatocyte invasion-inhibitory and complement-fixing activities (31).

Humoral reactivity to CSP is induced following CPS immunization (16, 17), but the induction of sporozoite-specific IgG does not correlate with anti-CSP-IgG levels. In fact, even the virtually complete depletion of anti-CSP-specific IgG (median percent CSP depletion, 91.7% [IQR, 79.8 to 96.4%]) (see Table S2 in the supplemental material) still leaves complement-fixing activity against sporozoites intact. This may suggest that only a fraction of anti-CSP-specific antibodies may still be sufficient for complement activation. It is more likely, however, that CPS-induced IgG antibodies with different antigenic specificities than CSP (17, 27) might show functional activity.

Showing some variation between volunteers, it is clear that CPS-induced antibodies can efficiently enhance C3 fixation, with subsequent enhanced homologous and heterologous sporozoite lysis. Complement fixation efficacy depends on antibody-intrinsic features, such as the epitope specificity of antibodies, antibody affinity as well as antibody isotype, or Fc receptor glycosylation. High-affinity antibodies can activate complement more efficiently, since antibodies may switch classes in a particular order depending on the degree of affinity maturation (32–35). As for antibody isotype, it is very likely that some individuals with low IgG concentrations may have high IgM levels, while other individuals might rely more on IgGs for complement-fixing activity. While CPS immunization predominantly induces anti-CSP-specific IgG1 and IgM antibodies, RTS,S (CSP) subunit vaccination primarily induces IgG1 antibodies against CSP repeats (36). On the other hand, CSP-specific naturally acquired antibodies are mainly IgG1, IgG3, and IgM (21), showing that the induction of anti-CSP antibody isotypes differs between these immunizations. A possible explanation for the absence of IgG2, IgG3, and IgG4 responses following CPS immunization might be that IgG subclass responses following whole-sporozoite immunization may be polarized toward IgG1, the most abundant immunoglobulin, and induced upon exposure to soluble protein antigens and membrane proteins (22). IgG2 and IgG4 antibody responses are produced only following exposure to bacterial polysaccharide antigens, in response to helminth infections, or following repeated or long-term exposure to noninfectious antigens (allergens) (37).

Alternatively, the observed variation in classical complement activation might be due to variation in the density of fragment crystallizable (Fc) regions, since a minimum threshold concentration of Fc regions is required for stronger classical pathway activation (34). Moreover, Fc receptor glycosylation can affect antigen-binding characteristics and, thus, antibody activity (22, 38).

As a next step, we studied the functional consequences of enhanced complement fixation on sporozoite infectivity *in vitro*. NF54 human hepatocyte invasion is inhibited by CPS-induced antibodies more strongly than is NF135.C10 invasion in the absence of active complement, as recently reported by us (26). Despite lower levels of IgM binding to NF135.C10 sporozoites, which is most likely due to genetic diversity in the CSP protein sequence (24), there were no differences in complement protein deposition and sporozoite lysis. This can be explained by the fact that IgM antibodies generally have a lower threshold for complement fixation than IgG antibodies and are therefore still very efficient in complement activation, despite the fact that they bind less efficiently to heterologous NF135.C10 sporozoites. Interestingly, the level of NF135.C10 invasion inhibition is twice as high as the level of NF54 inhibition in the presence of active complement. These data suggest that opsonization for complement might also occur independently of strain-specific epitopes and that complement has more added value when antibodies alone are less efficient in binding and neutralization, e.g., due to parasite genetic diversity. Antibody-mediated complement activation may also have additional indirect effects on sporozoite clearance, including modulation of the inflammatory response, induction of antibody-dependent cellular cytotoxicity (ADCC), or phagocytosis (39).

Unexpectedly, some C3 deposition and sporozoite death also take place before immunization, suggesting that some C3 complement proteins may bind directly to sporozoites via the alternative pathway. Some sporozoite proteins may contain carbohydrates that might activate the antibody-independent mannose-binding lectin (MBL) pathway (40–45). While MBL-deficient mice show no altered resistance to liver-stage infection, MBL binding may take place and modulate host defense (46). More likely, malaria-naive volunteers may have cross-reactive antibodies that recognize sporozoites or mosquito salivary gland material and thus are able to interact with complement proteins. Although possibly contributing, our data clearly show that complement activation is activated and functional primarily in the presence of malaria-specific antibodies. Additionally, it was recently shown that C3 deposition on CSP by naturally acquired antibodies strongly correlates with C1q fixation to CSP (21). Here, we show that NF54 invasion inhibition by CPS-induced antibodies and active complement is negatively associated with cumulative parasitemia during CPS immunizations. Previous studies in human and animal models of malaria support a role for complement activation during malaria infection with increased membrane attack complex (47) or reduced serum complement protein (48–53) levels. It was observed previously in a humanized liver-chimeric mouse model that CPS-induced antibodies had a much stronger inhibitory effect on P. falciparum liver-stage infection *in vivo* than *in vitro* (18). A possible explanation for this observation might be that CPS-induced IgGs could potentially interact with human complement, which was shown previously to be produced by human hepatocytes in human liver-chimeric mice (54), resulting in a more pronounced effect on liver-stage inhibition *in vivo*. Additionally, resistance to natural infection with Plasmodium malaria parasites has been associated with IgG1 and IgG3 antibody isotypes, both of which are very potent in fixing complement and activating the complement system (20, 55–57). This is further supported by the fact that children living in an area where P. falciparum is holoendemic in Papua New Guinea who have high levels of complement-fixing antibodies to CSP have a reduced risk of clinical malaria compared to children with undetectable functional antibodies (21). Taken together, data from these studies suggest that the complement system plays a role in antimalarial immunity and protection from malaria infection *in vivo*.

In summary, these findings demonstrate for the first time that CPS-induced antibodies can interact with the complement system, further reducing homologous and heterologous sporozoite infectivity *in vitro*. Together, these data highlight the importance of the complement pathway and provide new knowledge on antibody-mediated immune mechanisms involved in preerythrocytic immunity to homologous and heterologous sporozoites after whole-parasite immunization against P. falciparum malaria.

## MATERIALS AND METHODS

### Study design of experimental controlled human malaria infection.

Citrated plasma and serum samples were used from a double-blind, randomized, placebo-controlled CPS immunization trial conducted at the Radboud University Medical Center in 2015 (Nijmegen, The Netherlands) (ClinicalTrials.gov registration number NCT02098590) (24). Due to limited plasma sample availability, citrated plasma samples from a second open-labeled, randomized, CPS immunization study conducted in 2014 to 2015 (ClinicalTrials.gov registration number NCT02080026) were used. All study subjects provided written informed consent, and both studies were approved by the Central Committee for Research Involving Human Subjects of The Netherlands (CCMO) (approval numbers NL48732.091.14 and NL48301.091.14).

In both CPS trials (ClinicalTrials.gov registration numbers NCT02098590 and NCT02080026), volunteers were subjected to NF54 CPS immunization. While receiving chloroquine in a prophylactic dose, subjects were immunized three times (ClinicalTrials.gov registration number NCT02098590) or four times (ClinicalTrials.gov registration number NCT02080026) at monthly intervals by exposure to bites from 15 P. falciparum NF54-infected Anopheles stephensi mosquitoes. Fourteen weeks after the discontinuation of chloroquine prophylaxis, subjects underwent a primary challenge infection by exposure to bites of 5 mosquitoes infected with the homologous P. falciparum NF54 strain (58) or the genetically distinct NF135.C10 (23) and NF166.C8 (59) clones. Of note, study subjects from the second, open-label, randomized CPS immunization study were exposed only to NF54-infected mosquito bites (ClinicalTrials.gov registration number NCT02080026). All subjects were monitored closely from days 6 to 10 after each CPS immunization and from days 6 to 21 after mosquito bite challenge infection for symptoms and signs of malaria. When the treatment threshold (100 parasites per ml of blood) by quantitative PCR (qPCR) between days 7 and 9 was reached (60, 61), blood-stage parasitemia was treated with a curative regimen of atovaquone and proguanil once daily for 3 days. Cumulative blood-stage parasitemia during all three CPS immunizations (ClinicalTrials.gov registration number NCT02098590) was calculated by summing up the number of parasites per milliliter of blood, as determined by qPCR, from days 6 to 10 after each CPS immunization. NF54 CPS immunization induced sterile protection against NF54, NF135.C10, and NF166.C8 mosquito bite challenge infections in 5/5, 2/10, and 1/9 subjects, respectively. Six of ten NF135.C10-challenged subjects showed a prolonged prepatent period (ClinicalTrials.gov registration number NCT02098590). With respect to the second CPS immunization study (ClinicalTrials.gov registration number NCT02080026), 5/9 study subjects were completely protected from homologous NF54 mosquito bite challenge.

### Parasite strains.

P. falciparum NF54 and NF135.C10 sporozoites were used for *in vitro* sporozoite assays. Plasmodium falciparum NF54 was isolated from an individual near Schiphol Airport (The Netherlands) and most likely originated from West Africa (58, 62). The genetically and geographically distinct P. falciparum NF135.C10 clone originated from a clinical isolate in Cambodia (23).

### Parasite culture and generation of P. falciparum-infected mosquitoes.

P. falciparum NF54 and NF135.C10 asexual and sexual blood-stage parasites were cultured in a semiautomated culture system, as described previously (63–65). Anopheles stephensi mosquitoes were reared in the insectary of the Radboud University Medical Center, and female mosquitoes were infected by standard membrane feeding on NF54 or NF135.C10 gametocyte cultures (66). For *in vitro* sporozoite assays, salivary glands from infected mosquitoes were hand dissected 14 to 28 days after mosquito infection, collected in Leibovitz culture medium without serum, and homogenized in a homemade glass grinder. The number of P. falciparum NF54 or NF135.C10 sporozoites was determined with a Bürker-Türk counting chamber, using phase-contrast microscopy (18).

### Human hepatoma HC-04 cell line.

The HC-04 human hepatocyte cell line (Homo sapiens HC-04; MRA-965), deposited by Jetsumon Sattabongkot (67), was acquired through the Malaria Research and Reference Reagent Resource Center (MR4) as part of the Biodefense and Emerging Infections Research Resources Repository (BEI Resources). Cells were maintained in Dulbecco's modified Eagle medium (DMEM)–Ham's F-12 nutrient mixture medium (Gibco) supplemented with 10% heat-inactivated fetal bovine serum (FBS; Gibco), 1% glutamine (Gibco), and 1% penicillin-streptomycin (Gibco) at 37°C in an atmosphere of 5% CO_2_.

### Citrated plasma, serum samples, and complement source.

Citrated plasma samples and serum samples from 24 CPS-immunized volunteers were collected 11 to 14 days before the first CPS immunization (preimmunization) and 1 day before challenge infection (18 weeks after the last immunization) (postimmunization) (ClinicalTrials.gov registration number NCT02098590) by using citrated Vacutainer cell preparation tubes (CPT Vacutainers; Becton Dickinson) or serum Vacutainer tubes (Becton Dickinson). Samples were used for the determination of immunoglobulin subclasses, antibody opsonization assays (citrated plasma samples), or *in vitro* sporozoite assays (serum). Samples were stored in aliquots at −20°C. Prior to use in *in vitro* sporozoite assays, serum aliquots were heat inactivated for 30 min at 56°C, centrifuged at 13,000 rpm for 5 min at room temperature, and kept at 4°C.

Due to limited plasma availability, additional citrated plasma samples were collected 1 week before the first CPS immunization (preimmunization) and 1 day before challenge infection (15 weeks after the last immunization) (postimmunization) from 5 sterilely protected CPS-immunized volunteers (ClinicalTrials.gov registration number NCT02080026) and used for IgG purifications and depletion of CSP-specific antibodies. Purified IgGs were assessed for their ability to activate complement and induce sporozoite lysis in the presence or absence of CSP-specific antibodies.

An external source of human complement was used for all *in vitro* sporozoite assays to determine the complement-fixing activity of CPS-induced antibodies (either heat-inactivated CPS serum or purified IgGs) independently of possible differences in complement activity present in each CPS-immunized volunteer. This specific batch was consistently used for all experiments and samples and either added fresh (normal human serum [NHS]) (active complement) or heat inactivated (heat-inactivated serum [HIS]) (inactive complement). Heat inactivation was validated previously. This complement source consisted of pooled sera from 5 malaria-naive Australian donors and was validated for the absence of CSP-specific antibodies by a standardized enzyme-linked immunosorbent assay (ELISA), as described previously (21).

### Purification of IgG from citrated plasma samples.

Purification of IgG from citrated pre- and postimmunization plasma samples from CPS-immunized volunteers (*n* = 5) was performed by using a 5-ml HiTrap protein G HP affinity column (Amersham Biosciences) according to the manufacturer's instructions. A fraction of purified postimmunization IgGs was depleted from CSP-specific antibodies by running IgGs three times over a CSP affinity column that was constructed by coupling CSP (Gennova Biotechniques Pvt. Ltd., India) to a 1-ml HiTrap NHS-activated HP affinity column (catalog number 17-0717-01; GE Healthcare). IgGs were purified and depleted from CSP-specific antibodies by using an Äkta prime machine and Unicorn software (version 1.0; GE Healthcare). Purified IgGs were taken up in phosphate-buffered saline (PBS). IgG concentrations and CSP depletion efficacy (see Tables S1 and S2 in the supplemental material) were determined by standardized total IgG and CSP-IgG ELISAs prior to use in *in vitro* complement deposition and sporozoite damage assays.

### Total IgG, CSP-IgG, CSP-IgM, and CSP-specific Ig subclass ELISAs.

Total IgG concentrations and CSP-specific IgG antibodies in purified preimmunization IgGs, postimmunization IgGs, and CSP-depleted postimmunization IgGs and CSP-specific IgM antibody levels in pre- and postimmunization plasma samples were determined by total IgG, CSP-IgG, and CSP-IgM ELISAs. CPS-induced immunoglobulin isotypes to CSP were determined by CSP-specific Ig subclass ELISAs. All these ELISAs are described in detail in supplemental material.

### IgG and IgM antibody opsonization of whole sporozoites.

The recognition of whole sporozoites by immunization-induced IgG and IgM antibodies was determined by an *in vitro* flow cytometry-based antibody opsonization assay, as described in detail in the supplemental material. Briefly, 5 × 10^4^ P. falciparum NF54 or NF135.C10 sporozoites/well in a V-bottom 96-well plate were incubated with 10% heat-inactivated pre- or postimmunization serum and 10% heat-inactivated normal human serum (inactive complement) for 30 min at 37°C. Following incubation, samples were washed with PBS, centrifuged at 3,220 × *g* for 5 min at room temperature, and stained with fluorescently labeled antibodies targeting sporozoite CSP, IgG, and IgM for 30 min in the dark at 4°C. Unstained sporozoites and single compensation controls were included. Following incubation, samples were fixed with 1% paraformaldehyde (PFA) for 20 min in the dark at 4°C and taken up in PBS. Samples were kept at 4°C in the dark until flow cytometric analysis was performed. Flow cytometric analysis was performed with an LSRII flow cytometer (BD BioSciences), and data analysis was performed with FlowJo software (version 10.0.8; TreeStar).

### *In vitro* complement deposition and sporozoite damage assay.

C3 complement protein deposition on whole sporozoites and sporozoite damage due to complement activation in the presence or absence of immunization-induced antibodies were assessed with an *in vitro* flow cytometry-based assay, as described in detail in the supplemental material. Briefly, 5 × 10^4^ P. falciparum NF54 or NF135.C10 sporozoites/well in a V-bottom 96-well plate were incubated with 10% heat-inactivated pre- or postimmunization serum and 10% fresh normal human serum (active complement) or 10% inactive complement for 30 min at 37°C. In the case of purified IgGs, sporozoites were incubated with 10 mg/ml preimmunization IgGs, postimmunization IgGs, or postimmunization IgGs depleted from CSP in the presence of 10% active complement. Following incubation, PBS–20 mM EDTA was added to all samples, and plates were incubated at 4°C for 5 min to inactivate complement. Subsequently, sporozoites were stained with fluorescently labeled antibodies targeting sporozoite CSP, C3 complement protein deposition, and a fixable viability dye for 30 min in the dark at 4°C. Unstained sporozoites and single compensation controls were taken along. After incubation, samples were processed and analyzed as described above. The geometric mean fluorescence intensity (MFI) and the percentage of membrane-compromised sporozoites in postimmunization samples were corrected for those for preimmunization responses by subtracting the MFI and percentage of membrane-compromised sporozoites for preimmunization responses from that for postimmunization responses.

### *In vitro* sporozoite hepatocyte traversal inhibition assay.

The antibody-dependent complement-mediated effect of CPS-induced antibodies on further augmenting the inhibition of *in vitro* human hepatocyte traversal by P. falciparum sporozoites in the presence of active complement was assessed as described previously, with small adaptations (18). Briefly, freshly dissected NF54 and NF135.C10 sporozoites were preincubated with 10% heat-inactivated pre- or postimmunization CPS serum for 30 min at 4°C. Subsequently, 5 × 10^4^ sporozoites/well were added in duplicate to flat-bottom 96-well plates containing monolayers of 5 × 10^4^ HC-04 cells in the presence of 10% active or inactive complement and 0.5 mg/ml fixable tetramethylrhodamine dextran (Thermo Fisher Scientific). HC-04 cells alone in the presence of dextran served as a background control. After 2 h of incubation at 37°C in 5% CO_2_, cells were washed gently and processed for flow cytometric analysis. Flow cytometric analysis was performed with a cyan ADP flow cytometer (Beckman Coulter), and data were analyzed with FlowJo software (version 9.6.7; TreeStar). The percentage of dextran-positive cells was first corrected for background reactivity by subtracting the background, and the percent traversal inhibition was calculated as follows: 1 − (average percent dextran-positive cells in postimmunization cultures/average percent dextran-positive cells in preimmunization cultures) × 100%.

### *In vitro* sporozoite infectivity assay with a human hepatoma cell line.

The neutralization of P. falciparum sporozoite hepatocyte invasion *in vitro* by CPS-induced antibodies was assessed by a flow cytometry-based *in vitro* invasion assay, as described in detail in the supplemental material. Briefly, P. falciparum NF54 or NF135.C10 sporozoites were preincubated with 10% heat-inactivated pre- or postimmunization CPS serum for 30 min at 4°C. Subsequently, 5 × 10^4^ sporozoites/well were added to flat-bottom 96-well plates containing monolayers of 5 × 10^4^ HC-04 cells in the presence of 10% active or inactive complement. After 3 h of incubation at 37°C in 5% CO_2_, intracellular and invaded parasites were stained with a fluorescently labeled antibody targeting CSP, and cells were processed for flow cytometric analysis. Flow cytometric analysis was performed with a Gallios flow cytometer (Beckman Coulter), and data were analyzed with FlowJo software (version 10.0.8; TreeStar). The percentage of CSP-positive sporozoites was first corrected for background reactivity by subtracting the background (uninfected HC-04 cells in the presence of 3SP2-Alexa Fluor 488 antibody). The percent invasion inhibition was calculated as follows: 1 − (average percent CSP-positive cells in postimmunization cultures/average percent CSP-positive cells in preimmunization cultures) × 100%.

### Statistical analysis.

Statistical analysis was performed by using GraphPad Prism software (version 5; GraphPad Software Inc., CA, USA). For analysis of *in vitro* sporozoite data, antibody binding, C3 complement deposition on sporozoites, and sporozoite lysis, differences between pre- and postimmunization samples, HIS and NHS, or parasite strains were tested by using two-tailed paired Student's *t* test. Comparisons of two nonmatching groups (controls versus CPS-immunized volunteers) or comparisons between multiple groups (pre- and postimmunization IgGs and CSP-depleted postimmunization IgGs) were tested with unpaired Student's *t* test or one-way analysis of variance (ANOVA) with a Bonferroni multiple-comparison *post hoc* test, respectively. Correlation analyses were conducted with Spearman correlation analysis. A *P* value of <0.05 was considered significant.

## Supplementary Material

Supplemental material
