# Supplementary material for: The Complement System Contributes to Functional Antibody-Mediated Responses Induced by Immunization with Plasmodium falciparum Malaria Sporozoites
Source: Infect Immun. 2018 Jun 21;86(7):e00920-17. doi: 10.1128/IAI.00920-17 (PMC6013677; doi:10.1128/IAI.00920-17)
Supplement: Supplemental material [file supp_86_7_e00920-17__index.html]

Supplemental material 

# The Complement System Contributes to Functional Antibody-Mediated Responses Induced by Immunization with Plasmodium falciparum Malaria Sporozoites

## Supplemental material

- Supplemental file 1 -

  Supplemental materials and methods.

  PDF, 468K
- Supplemental file 2 -

  Legends for Fig. S1 to S4.

  PDF, 342K
- Supplemental file 3 -

  Table S1. Total IgG concentrations.

  PDF, 175K
- Supplemental file 4 -

  Table S2. CSP depletion efficacy.

  PDF, 177K
- Supplemental file 5 -

  Fig. S1. Induction of IgG and IgM sporozoite-specific antibodies in control versus CPS-immunized volunteers.

  TIF, 747K
- Supplemental file 6 -

  Fig. S2. Antibody specificity of CPS-induced sporozoite-specific IgG and IgM antibodies.

  TIF, 919K
- Supplemental file 7 -

  Fig. S3. Importance of antibody-independent pathways for complement activation and sporozoite lysis.

  TIF, 837K
- Supplemental file 8 -

  Fig. S4. Correlation analysis of invasion inhibition and cumulative parasitemia or prepatent period.

  TIF, 505K
