# Supplementary material for: The Complement System Contributes to Functional Antibody-Mediated Responses Induced by Immunization with Plasmodium falciparum Malaria Sporozoites
Source: Infect Immun. 2018 Jun 21;86(7):e00920-17. doi: 10.1128/IAI.00920-17 (PMC6013677; doi:10.1128/IAI.00920-17)
Supplement: Supplemental material [file IAI.00920-17_zii999092463s1.pdf]

Supplemental Materials and Methods

Total IgG ELISA

Total IgG concentrations in purified pre-immunization IgGs, post-immunization IgGs and CSP-depleted post-immunization IgGs were determined by total IgG-ELISAs, as described below (*see CSP-IgG ELISA*) with small adaptations. Briefly, polystyrene flat-bottom 96-well plates were coated overnight with 1 ug/ml goat anti-human IgG (ImmunoPure, ThermoFischer Scientific). Purified IgGs were diluted 1:2,000 in PBS containing 0.05% Tween20 and 1% milk (PBST/1% milk) and a seven-point 1:3 dilution series was carried out for each IgG sample. As a standard and positive control, purified malaria-naive IgGs with a fixed concentration (5.7 mg/ml) were diluted 1:2,000 in PBST/1% milk and included on every plate in a seven-point 1:3 dilution series in duplicate. IgG concentrations were calculated in relation to the positive control.

CSP-IgG ELISA

Levels of *P. falciparum* circumsporozoite protein (CSP)-specific antibodies in purified IgGs were determined by ELISAs as previously described with small adaptations (8). Briefly, polystyrene flat-bottom 96-well plates were coated overnight with 1 ug/ml CSP in PBS (Gennova Biotechniques Pvt. Ltd., India). Purified IgG samples were diluted 1:50 in PBST/1% milk and a seven-point 1:2 dilution series was carried out for each IgG sample. As a standard and positive control, a plasma pool from 100 Tanzanian hyper-immune adults living in a highly malaria-endemic area (HIT) was diluted 1:200 in PBST/1% milk and included on every plate in a seven-point 1:2 dilution series in duplicate.

CSP-IgM ELISA

Levels of *P. falciparum* circumsporozoite protein (CSP)-specific IgM antibodies were determined by ELISAs that have previously been optimized for type of blocking buffer, concentrations of IgM horseradish peroxidase (HRP) detection antibodies and plasma sample dilutions. Polystyrene flat-bottom 96-well plates were coated overnight with 0.5 ug/ml CSP in PBS (Gennova Biotechniques Pvt. Ltd., India). Plates were washed four times prior to blocking with PBS/3% BSA for one hour at room temperature. Citrated pre- and post-immunization plasma samples were diluted 1:50 in PBS containing 0.05% Tween20 and 1% BSA (PBST/1% BSA) and a four-point 1:3 dilution series was carried out for each sample. For CSP-IgM ELISAs the same standard and positive control was used as for CSP-IgG ELISAs; HIT serum was diluted 1:200 in PBST/1% milk and included on every plate in a seven-point 1:2 dilution series in duplicate. After a three hour incubation step at room temperature, plates were washed four times with PBS. Goat anti-human IgM-HRP detection antibody (Dako; P0215) diluted 1/6,250 in PBST was added to each well and after an one hour incubation step, plates were washed four times with PBS. Bound IgG and IgM antibodies were detected with a horseradish peroxidase (HRP)-conjugated detection antibody, followed by development with HRP substrate (tetramethylbenzidine). HRP substrate development was stopped by the addition of 0.2M sulfuric acid (H_2SO_4).

CSP-specific Ig subclass ELISA

Polystyrene flat-bottom 96-well plates were coated overnight at 4 °C with 0.5 ug/ml CSP in PBS (Gennova Biotechniques Pvt. Ltd., India). Plates were washed three times with PBST (PBS containing 0.05% Tween20), blocked for 2 hours at 37 °C with 10% milk in PBS, and

again washed three times with PBST. Pre- and Post-immunization plasma samples from CPS-immunized volunteers (n=15) were diluted 1:100 in PBST/5% milk and a four-point 1:2 dilution series was carried out for each sample. As a standard and positive control, HIT was diluted 1:100 in PBST/1% milk and included on every plate in a seven-point 1:2 dilution series in duplicate. Plasma samples were incubated for 2 hours at room temperature and subsequently washed three times with PBST. IgG subclass antibodies recognizing CSP were detected by adding 1:1,000 dilution of biotinylated IgG1 (MabTech, 3851-6-250), IgG2 (MabTech, 3852-6-250), IgG3 (MabTech, 3853-6-250) or IgG4 (MabTech, 3854-6-250) antibodies for 1 hour at room temperature. Following a wash step with PBST, streptavidin-HRP (MabTech, 3310-9) was added to each well at a 1:1,000 dilution. Wells were washed three times with PBST, followed by three times with PBS, and developed with HRP substrate (tetra-methyl-benzidine). The reaction was stopped with 0.2M sulfuric acid. As a technical control for the detection of IgG1, IgG2, IgG3 and IgG4 antibodies, polystyrene flat-bottom 96-well plates were coated overnight at 4 °C with 1:50 dilution of a malaria-naive donor, followed by detection steps as described in the previous section.

For all ELISAs as described above, all samples from each volunteer were measured on the same plate. Spectrophotometrical absorbance at 450 nm was measured using the iMark Microplate Absorbance Reader (Bio-Rad). Antibody levels were expressed as arbitrary units (AU) in relation to the positive control, set at 100 AU (11). ELISA data analysis was performed with Auditable Data Analysis and Management System for ELISA (ADAMSEL, version 1.1) as previously described (16).

IgG and IgM antibody opsonization of whole sporozoites

P. falciparum NF54 or NF135.C10 sporozoites collected in Leibovitz culture medium were added to v-bottom 96-well plates (CoStar) at a concentration of $5x10^4$ sporozoites/well and PBS was topped up to 200 ul/well. Samples were centrifuged at 3,220 g for 5 minutes at room temperature (Eppendorf Centrifuge 5810 R). Sporozoites were incubated with 10% heat-inactivated pre- or post-immunization serum and 10% heat-inactivated normal human serum (inactive complement) diluted in PBS for 30 minutes at 37 ˚C in duplicate (50 ul/well with 20% total serum in each well). Unstained sporozoites served as an internal control and were incubated in PBS alone. Other single compensation controls were incubated in the presence of 10% heat-inactivated post-immunization serum and 10% inactive complement. Following incubation, samples were washed with PBS and centrifuged at 3,220 g for 5 minutes at room temperature. Subsequently, sporozoites were stained with fluorescently labeled antibodies targeting sporozoite circumsporozoite protein (CSP; 3SP2-Alexa Fluor-488 at 1:1,000 dilution, labeled in house), IgG antibodies (IgG-PerCP at 1:50 dilution, Jackson ImmunoResearch laboratories) and IgM antibodies (IgM-Alexa Fluor-647 at 1:1,000 dilution, Jackson ImmunoResearch laboratories) diluted in PBS/2% bovine serum albumin (BSA), for 30 minutes in the dark at 4 ˚C. Unstained sporozoites were taken up in PBS/2% BSA and single compensation controls were taken along (sporozoites stained with only one single antibody; 3SP2-Alexa Fluor-488, IgG-PerCP or IgM-Alexa Fluor-647). After incubation, samples were washed with PBS, centrifuged at 3,220 g for 5 minutes at room temperature, and subsequently fixed with 1% paraformaldehyde (PFA) for 20 minutes in the dark at 4 ˚C. Lastly, samples were washed with PBS, centrifuged at 3,220 g for 5 minutes at room temperature and resuspended in 200 ul PBS. Samples were kept at 4 °C in the dark until flow cytometric analysis. Flow cytometric analysis was performed with a LSRII flow cytometer (BD

BioSciences) and data analysis was performed with FlowJo software (version 10.0.8, Tree Star).

In vitro complement deposition and sporozoite damage assay

P. falciparum NF54 or NF135.C10 sporozoites collected in Leibovitz culture medium were added to v-bottom 96-well plates (CoStar) at a concentration of $5x10^4$ sporozoites/well and PBS was topped up to 200 ul/well. Plates were centrifuged at 3,220 g for 5 minutes at room temperature. Sporozoites were incubated with 10% heat-inactivated pre- of post-immunization serum and 10% fresh normal human serum (active complement) or 10% heat-inactive complement diluted in 1x veronal buffer (Lonza BioWhittaker) for 30 minutes at 37 ˚C in duplicate (total serum in each well: 20%). In case of purified IgGs, sporozoites were incubated with 10 mg/ml pre-immunization IgGs, post-immunization IgGs or post-immunization IgGs depleted from CSP in the presence of 10% active complement and diluted in 1x veronal buffer, for 30 minutes at 37 °C in duplicate. Unstained sporozoites served as an internal control and were incubated in 1x veronal buffer. Other (single) compensation controls were incubated in the presence of 10% heat-inactivated post-immunization serum or 10 mg/ml post-immunization IgGs and 10% active complement in 1x veronal buffer. To inactivate complement, PBS/20 mM EDTA was added 1:1 to all samples (final concentration: PBS/10 mM EDTA) and plates were put at 4 ˚C for 5 minutes to inactivate complement. Subsequently, samples were washed with PBS and centrifuged at 3,220 g for 5 minutes at room temperature. Sporozoites were then stained with fluorescently labeled antibodies targeting sporozoite CSP (3SP2-Alexa Fluor-647 at 1:10,000 dilution), C3 complement protein deposition (C3-FITC at 1:500 dilution; Cappel, MP Biomedicals) and a fixable viability dye (eFluor 450 at 1:4,000 dilution; eBioscience) diluted in PBS/2% BSA, for 30 minutes in the

dark at 4 ˚C. Unstained sporozoites were resuspended in PBS/2% BSA and single compensation controls were taken along: sporozoites stained with 3SP2-Alexa Fluor-647, C3-FITC or eFluor 450 detection antibodies. After incubation, samples were processed and analysed as described in the previous section. The geometric mean fluorescent intensity (MFI) and percent membrane-compromised sporozoites of post-immunization samples were corrected for that of pre-immunization responses by subtracting the MFI and percent membrane-compromised cells of pre-immunization responses from that of post-immunization responses.

In vitro sporozoite infectivity assay of a human hepatoma cell line

Neutralization of *P. falciparum* sporozoite hepatocyte invasion *in vitro* by CPS-induced antibodies was assessed in a flow-cytometry based *in vitro* invasion assay. Freshly dissected *P. falciparum* NF54 or NF135.C10 sporozoites were pre-incubated with 10% heat-inactivated pre- or post-immunization CPS serum for 30 minutes at 4 ˚C. Subsequently, $5x10^4$ sporozoites/well were added in duplicate to flat-bottom 96-well plates containing monolayers of $5x10^4$ HC-04 cells in the presence of 10% active or inactive complement. Samples were centrifuged at 3,000 rpm for 10 minutes at room temperature with a low brake and after 3 hours of incubation at 37 °C in 5% CO_2, cells were gently washed three times with PBS to remove excess sporozoites and antibodies. Cells were trypsinized with 0.05% Trypsin-EDTA for a couple of minutes at room temperature and trypsin activity was subsequently neutralized by adding 10% FBS in PBS. All cells were transferred to a 96-well v-bottom plate and centrifuged at 1,700 rpm for 4 minutes at 4°C. Supernatant was carefully removed, samples were fixed and permeabilized with Fixation/Permeabilization buffer (eBioscience) for 30 minutes at 4 °C. Subsequently, samples were washed with

Permeabilization buffer (eBioscience) and centrifuged at 1,700 rpm for 4 minutes at 4 °C. Intracellular and invaded parasites were stained with an anti-CSP monoclonal antibody conjugated with fluorescent Alexa Fluor 488 (3SP2-Alexa Fluor-488; diluted 1:160 in 10% FBS in PBS), and incubated for 30 minutes in the dark at 4 °C. Samples were again washed with Permeabilization buffer and pellets were taken up in 1% PFA, transferred to micronic tubes (Micronic), and put at 4 °C in the dark until flow cytometric analysis on the same day. Flow cytometric analysis was performed with a Gallios (Beckman Coulter) flow cytometer and data were analysed with FlowJo software (version 10.0.8, Tree Star). The percentage of CSP-positive sporozoites was first corrected for background reactivity by subtracting the background (uninfected HC-04 cells in the presence of 3SP2-Alexa Fluor-488 antibody). The percent invasion inhibition was calculated as follows: 1 - (average % CSP-positive cells in post-immunization cultures / average % CSP-positive cells in pre-immunization cultures) * 100%.

References

1. Delemarre BJ, van der Kaay HJ. 1979. [Tropical malaria contracted the natural way in the Netherlands]. Ned Tijdschr Geneeskd 123:1981-2.
2. Teirlinck AC, Roestenberg M, van de Vegte-Bolmer M, Scholzen A, Heinrichs MJ, Siebelink-Stoter R, Graumans W, van Gemert GJ, Teelen K, Vos MW, Nganou-Makamdop K, Borrmann S, Rozier YP, Erkens MA, Luty AJ, Hermsen CC, Sim BK, van Lieshout L, Hoffman SL, Visser LG, Sauerwein RW. 2013. NF135.C10: a new Plasmodium falciparum clone for controlled human malaria infections. J Infect Dis 207:656-60.
3. McCall MBB, Wammes LJ, Langenberg MCC, van Gemert GJ, Walk J, Hermsen CC, Graumans W, Koelewijn R, Franetich JF, Chishimba S, Gerdsen M, Lorthiois A, van de Vegte M, Mazier D, Bijker EM, van Hellemond JJ, van Genderen PJJ, Sauerwein RW. 2017. Infectivity of Plasmodium falciparum sporozoites determines emerging parasitemia in infected volunteers. Sci Transl Med 9.
4. Hermsen CC, Telgt DS, Linders EH, van de Locht LA, Eling WM, Mensink EJ, Sauerwein RW. 2001. Detection of Plasmodium falciparum malaria parasites in vivo by real-time quantitative PCR. Mol Biochem Parasitol 118:247-51.
5. Walk J, Schats R, Langenberg MC, Reuling IJ, Teelen K, Roestenberg M, Hermsen CC, Visser LG, Sauerwein RW. 2016. Diagnosis and treatment based on quantitative PCR after controlled human malaria infection. Malar J 15:398.
6. Roestenberg M, McCall M, Hopman J, Wiersma J, Luty AJ, van Gemert GJ, van de Vegte-Bolmer M, van Schaijk B, Teelen K, Arens T, Spaarman L, de Mast Q, Roeffen W, Snounou G, Renia L, van der Ven A, Hermsen CC, Sauerwein R. 2009. Protection against a malaria challenge by sporozoite inoculation. N Engl J Med 361:468-77.
7. Nahrendorf W, Scholzen A, Bijker EM, Teirlinck AC, Bastiaens GJ, Schats R, Hermsen CC, Visser LG, Langhorne J, Sauerwein RW. 2014. Memory B-cell and antibody responses induced by Plasmodium falciparum sporozoite immunization. J Infect Dis 210:1981-90.
8. Walk J, Reuling IJ, Behet MC, Meerstein-Kessel L, Graumans W, van Gemert GJ, Siebelink-Stoter R, van de Vegte-Bolmer M, Janssen T, Teelen K, de Wilt JHW, de Mast Q, van der Ven AJ, Diez Benavente E, Campino S, Clark TG, Huynen MA, Hermsen CC, Bijker EM, Scholzen A, Sauerwein RW. 2017. Modest heterologous protection after Plasmodium falciparum sporozoite immunization: a double-blind randomized controlled clinical trial. BMC Med 15:168.
