# Supplementary material for: The Complement System Contributes to Functional Antibody-Mediated Responses Induced by Immunization with Plasmodium falciparum Malaria Sporozoites
Source: Infect Immun. 2018 Jun 21;86(7):e00920-17. doi: 10.1128/IAI.00920-17 (PMC6013677; doi:10.1128/IAI.00920-17)
Supplement: Supplemental material [file IAI.00920-17_zii999092463s2.pdf]

Supplemental Information captions and legends

Figure S1: Induction of IgG and IgM sporozoite-specific antibodies in control *versus* CPS-immunized volunteers

Homologous NF54 sporozoites were pre-incubated with 10% heat-inactivated pre- or post-immunization serum from control volunteers and 10% inactive complement. Recognition of homologous NF54 sporozoites by IgG **(A)** and IgM **(B)** antibodies from control volunteers (n=10) all of which were under chloroquine prophylaxis while exposed to uninfected mosquito bites instead of infected mosquito bites. Differences in IgG **(C)** and IgM **(D)** antibody recognition of NF54 sporozoites by control volunteers (n=10) and CPS-immunized volunteers (n=16) are shown. Data are shown as the mean of duplicate measurements in MFI. Differences between pre- and post-immunization serum were tested using the paired Student's t-test, while differences between controls and CPS-immunized volunteers were tested using the unpaired Student's t-test. Data were considered statistically significant at $p<0.05$.

Figure S2: Antibody specificity of CPS-induced sporozoite-specific IgG and IgM antibodies

(A) Scatter plot of sporozoite-specific IgG and IgM antibodies targeting homologous NF54 sporozoites is shown (n=16 CPS-immunized volunteers). **(B)** Scatter plot of CSP-IgG and CSP-IgM antibodies is shown (n=16 CPS-immunized volunteers). **(C)** Correlation analysis for NF54 sporozoite-specific and CSP-specific IgG antibodies as previously determined by ELISAs (23) is shown for n=16 volunteers. **(D)** Correlation analysis for NF54 sporozoite-specific and CSP-specific IgM antibodies is shown (n=16 CPS-immunized volunteers). Data are shown as the

mean of duplicate measurements. Correlation analyses were conducted using Spearman correlation analysis. Data were considered statistically significant at $p<0.05$.

Figure S3: Importance of antibody-independent pathways for complement activation and sporozoite lysis

Activation of the complement pathway via antibody-independent pathways was characterized by looking at C3 complement protein deposition on homologous NF54 sporozoites **(A)** or NF54 sporozoite lysis **(B)** in the presence of malaria-naive (pre-immunization) antibodies (n=12 volunteers) in the presence of active (NHS) or inactive (HIS) complement. To show that homologous complement activation was more strongly induced by post-immunization antibodies, C3 deposition **(C)** and sporozoite lysis **(D)** by CPS-induced antibodies in the presence of active complement, compared to pre-immunization antibodies, are shown. Antibody-independent complement activation of heterologous NF135.C10 sporozoites was shown as C3 deposition. Data are shown as the mean of duplicate measurements. Differences between parasite strains (matched volunteers) were tested using the paired Student's t-test. Data were considered statistically significant at $p<0.05$.

Figure S4: Correlation analysis of invasion inhibition and cumulative parasitaemia or prepatent period

(A) Spearman correlation analysis between cumulative parasitaemia (*P. falciparum* NF54 parasites per milliliter) during three CPS-immunizations and NF54 invasion inhibition by CPS-induced antibodies in the presence of inactive complement (uncorrected for HIS; n=17 volunteers). **(B)** Spearman correlation analysis between NF135.C10 pre-patent period (days after challenge) and NF135.C10 invasion inhibition by CPS-induced antibodies in the

presence of active complement (n=4 volunteers). Data were considered statistically significant at $p<0.05$.

Table S1: Total IgG concentrations

Table S2: CSP depletion efficacy
