# Supplementary material for: The Complement System Contributes to Functional Antibody-Mediated Responses Induced by Immunization with Plasmodium falciparum Malaria Sporozoites
Source: Infect Immun. 2018 Jun 21;86(7):e00920-17. doi: 10.1128/IAI.00920-17 (PMC6013677; doi:10.1128/IAI.00920-17)
Supplement: Supplemental material [file IAI.00920-17_zii999092463s3.pdf]

Volunteer ID	Pre-immunization IgG (mg/ml)	Post-immunization IgG (mg/ml)	CSP-depleted post-immunization IgG (mg/ml)
**1**	44.4	15.5	27.7
**2**	15.1	101.3	36.7
**3**	39.2	29.9	9.7
**4**	16.4	16.7	13.1
**5**	15.7	42.9	14.8

Table S1: Total IgG concentrations
