# Supplementary material for: The Complement System Contributes to Functional Antibody-Mediated Responses Induced by Immunization with Plasmodium falciparum Malaria Sporozoites
Source: Infect Immun. 2018 Jun 21;86(7):e00920-17. doi: 10.1128/IAI.00920-17 (PMC6013677; doi:10.1128/IAI.00920-17)
Supplement: Supplemental material [file IAI.00920-17_zii999092463s4.pdf]

Volunteer ID	Post-immunization IgG (HIT=100 AU)	CSP-depleted post-immunization IgG (HIT=100 AU)	Percent CSP depleted from post-immunization IgG (%)
**1**	200.7	39.4	80.4
**2**	1316.0	45.5	96.5
**3**	175.5	14.5	91.7
**4**	25.9	5.4	79.2
**5**	1339.4	50.7	96.2

Table S2: CSP depletion efficacy
