# Supplementary figures and images for: The Complement System Contributes to Functional Antibody-Mediated Responses Induced by Immunization with Plasmodium falciparum Malaria Sporozoites
Source: Infect Immun. 2018 Jun 21;86(7):e00920-17. doi: 10.1128/IAI.00920-17 (PMC6013677; doi:10.1128/IAI.00920-17)

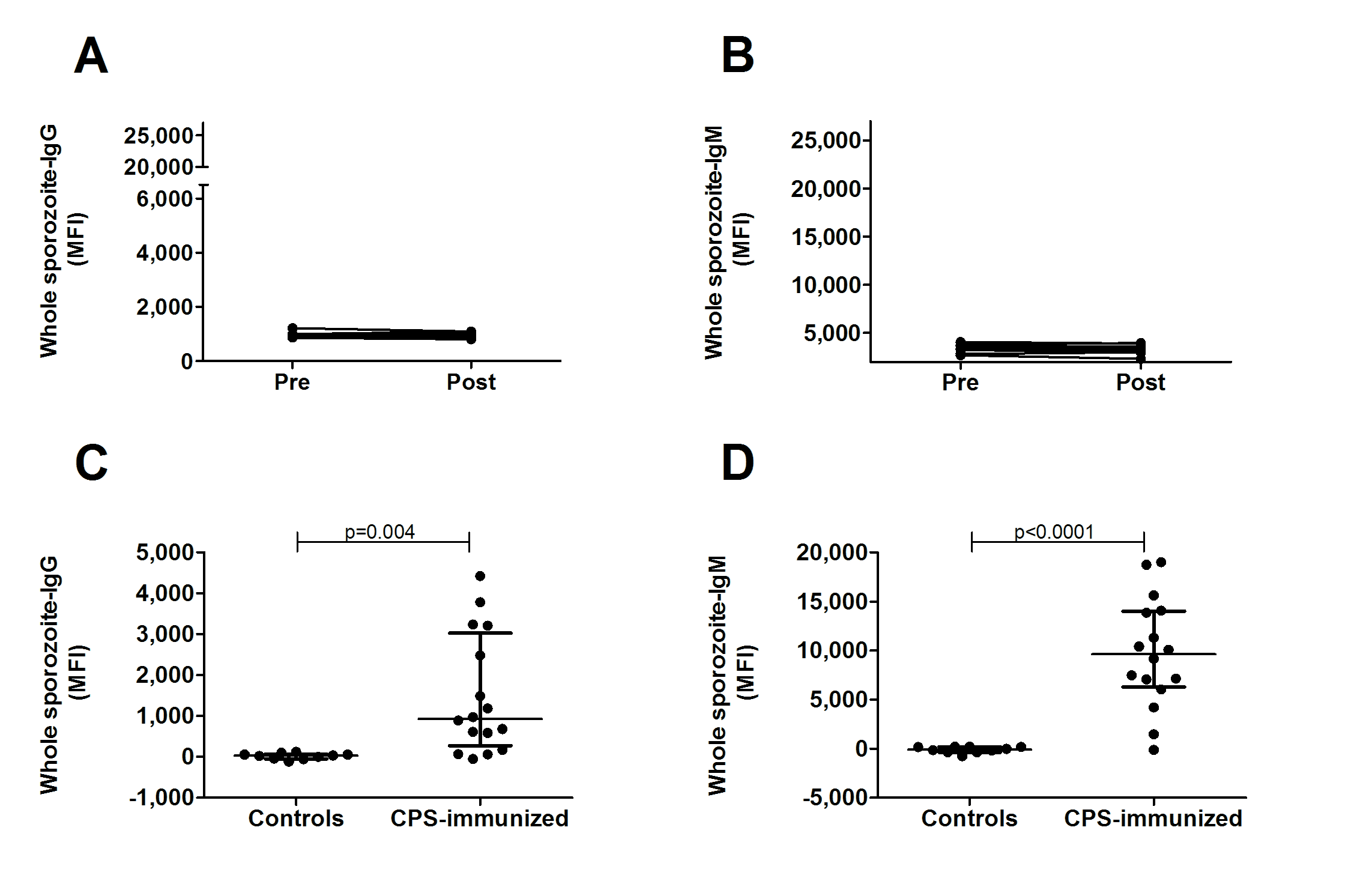

Supplement: Supplemental material [file IAI.00920-17_zii999092463s5.tif]

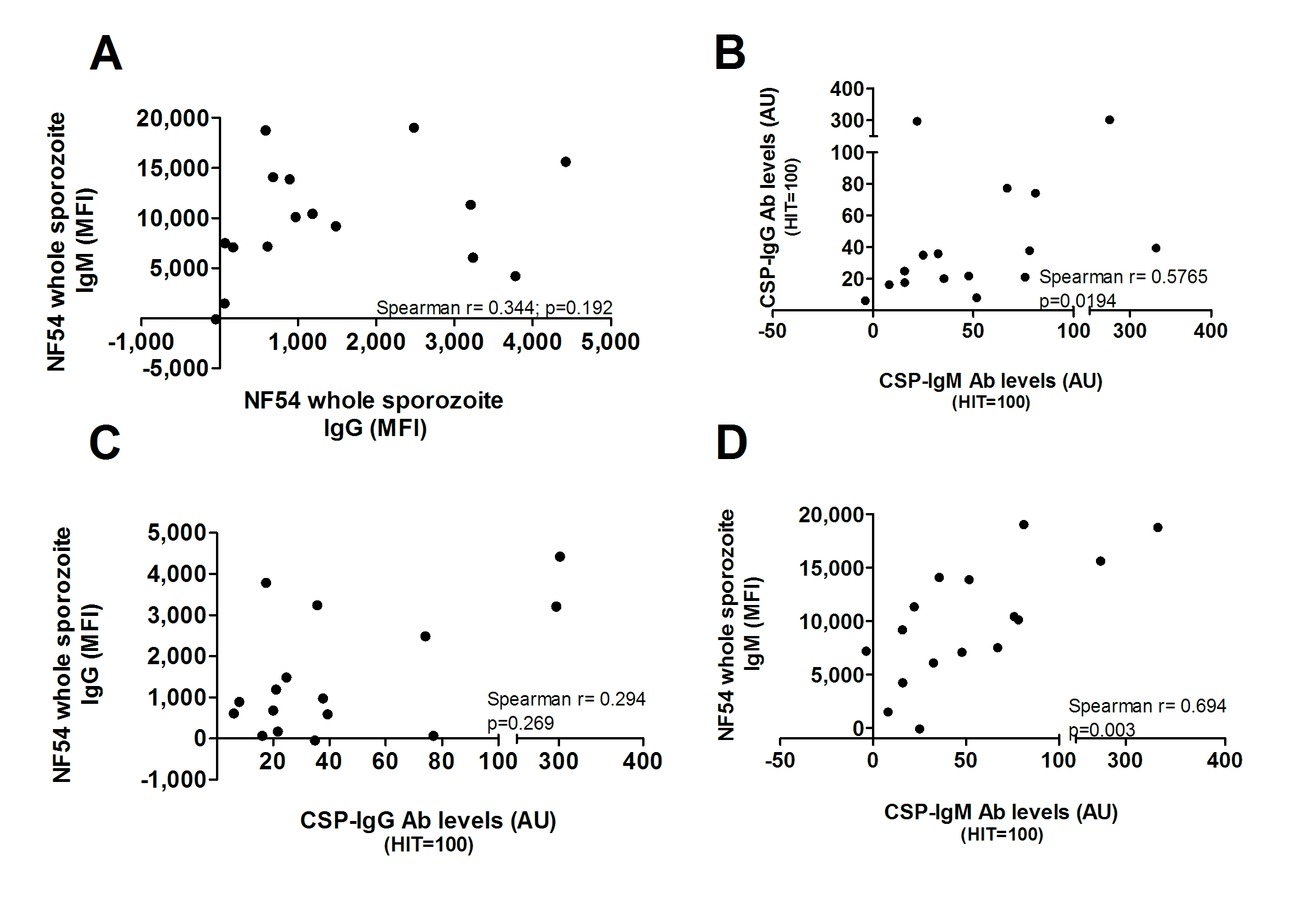

Supplement: Supplemental material [file IAI.00920-17_zii999092463s6.tif]

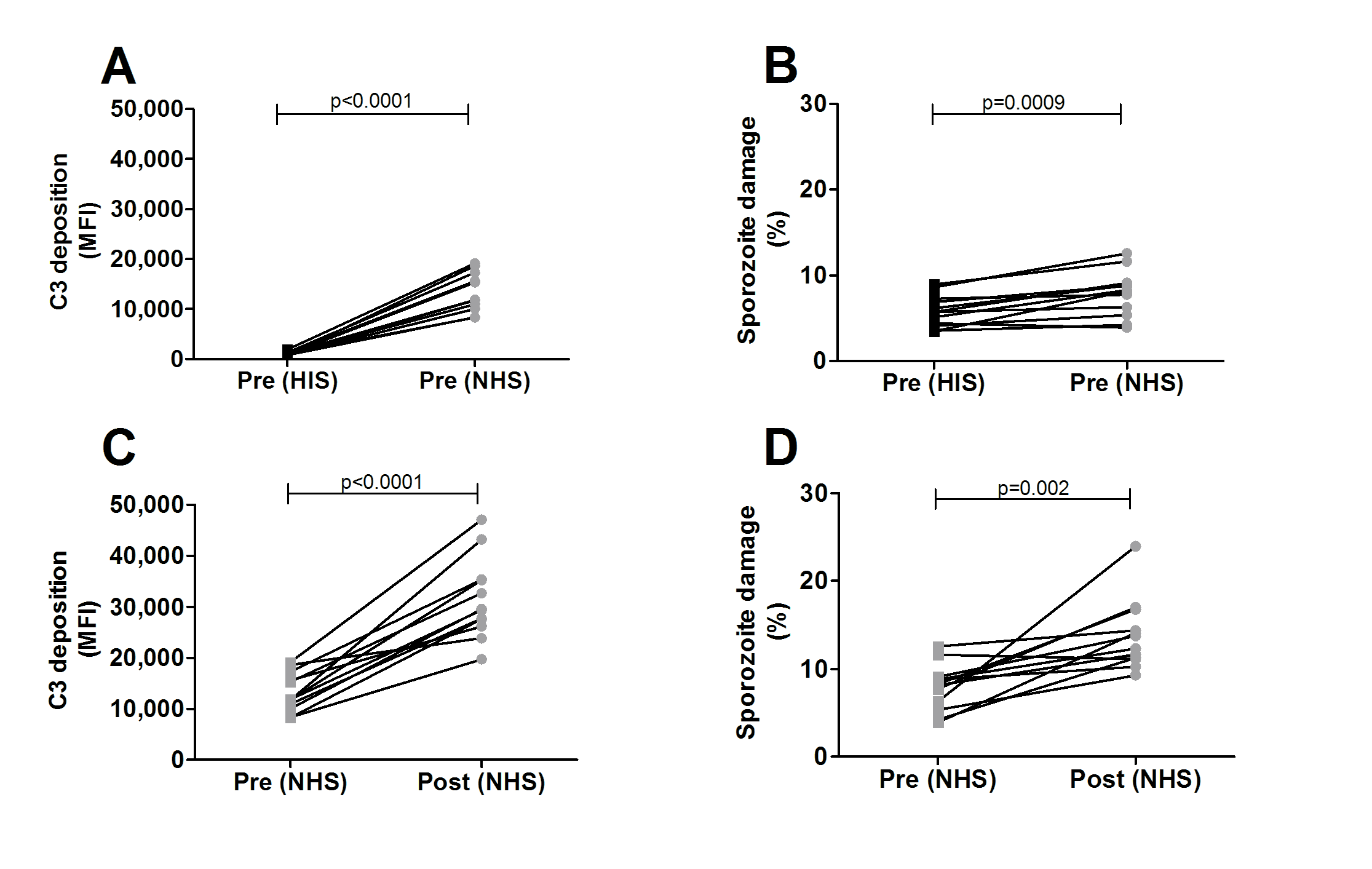

Supplement: Supplemental material [file IAI.00920-17_zii999092463s7.tif]

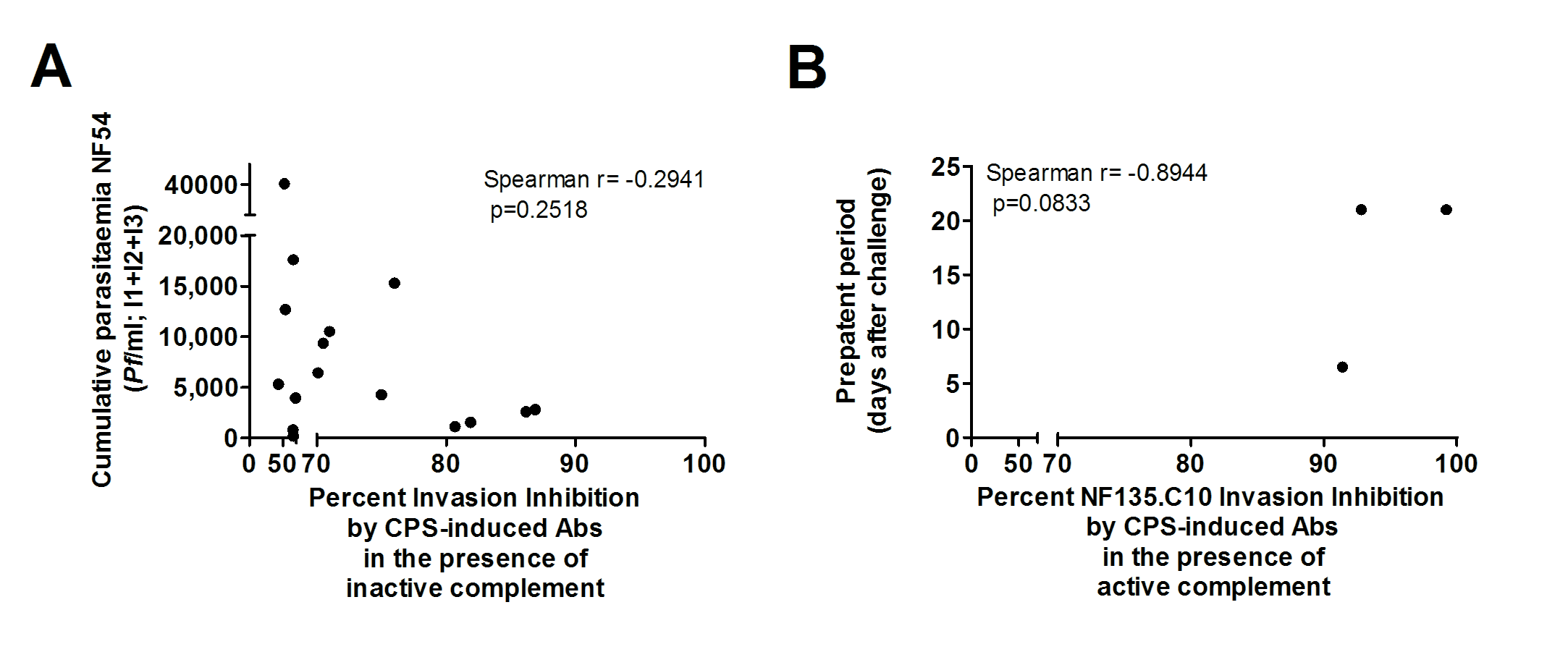

Supplement: Supplemental material [file IAI.00920-17_zii999092463s8.tif]
